# MFF-Net: A Lightweight Multi-Frequency Network for Measuring Heart Rhythm from Facial Videos

**DOI:** 10.3390/s24247937

**Published:** 2024-12-12

**Authors:** Wenqin Yan, Jialiang Zhuang, Yuheng Chen, Yun Zhang, Xiujuan Zheng

**Affiliations:** 1 College of Electrical Engineering, Sichuan University, Chengdu 610065, China; yanwenqin@stu.scu.edu.cn (W.Y.); jialiangzhuang97@163.com (J.Z.); 2021223035109@stu.scu.edu.cn (Y.C.); 2 Key Laboratory of Information and Automation Technology of Sichuan Province, Chengdu 610065, China; 3 School of Information Science and Technology, Xi’an Jiaotong University, Xi’an 710049, China; zhangyun2011@xjtu.edu.cn

**Keywords:** remote photo-plethysmography, lightweight, heart rate, heart rate variability, over-sampling training strategy

## Abstract

Remote photo-plethysmography (rPPG) is a useful camera-based health motioning method that can measure the heart rhythm from facial videos. Many well-established deep learning models can provide highly accurate and robust results in measuring heart rate (HR) and heart rate variability (HRV). However, these methods are unable to effectively eliminate illumination variation and motion artifact disturbances, and their substantial computational resource requirements significantly limit their applicability in real-world scenarios. Hence, we propose a lightweight multi-frequency network named MFF-Net to measure heart rhythm via facial videos in a short time. Firstly, we propose a multi-frequency mode signal fusion (MFF) mechanism, which can separate the characteristics of different modes of the original rPPG signals and send them to a processor with independent parameters, helping the network recover blood volume pulse (BVP) signals accurately under a complex noise environment. In addition, in order to help the network extract the characteristics of different modal signals effectively, we designed a temporal multiscale convolution module (TMSC-module) and spectrum self-attention module (SSA-module). The TMSC-module can expand the receptive field of the signal-refining network, obtain more abundant multiscale information, and transmit it to the signal reconstruction network. The SSA-module can help a signal reconstruction network locate the obvious inferior parts in the reconstruction process so as to make better decisions when merging multi-dimensional signals. Finally, in order to solve the over-fitting phenomenon that easily occurs in the network, we propose an over-fitting sampling training scheme to further improve the fitting ability of the network. Comprehensive experiments were conducted on three benchmark datasets, and we estimated HR and HRV based on the BVP signals derived by MFF-Net. Compared with state-of-the-art methods, our approach achieves better performance both on HR and HRV estimation with lower computational burden. We can conclude that the proposed MFF-Net has the opportunity to be applied in many real-world scenarios.

## 1. Introduction

Blood volume pulse (BVP) is an essential physiological signal with great potential in healthcare monitoring. Although many excellent works have proven that the contact-based method can comprehensively and accurately measure cardiac activities, the sensors attached to the human body cause discomfort and inconvenience. In recent years, an increasing number of methods have been proposed for measuring heart rate (HR) and BVP signals using video or microwave sensing. In this field, remote photo-plethysmography (rPPG) is a fast-growing technique to measure heart rhythm from facial videos. The unsupervised signal processing algorithms, including independent component analysis (ICA) [1], empirical mode decomposition (EMD) [2], and variational mode extraction(VME) [3], were proposed to derive the effective compont of signals to measure heart rhythm. Nevertheless, these methods are limited by poor generalization, which makes it difficult for them to deal with complex noise due to the changing ambient light conditions and face movements.

In this context, deep learning is increasingly recognized as a superior approach to overcome these problems. The robust semantic feature extraction capabilities of neural networks enable them to effectively learn from and adapt to complex noise environments. As a result, numerous deep learning-based methods for HR computation have emerged, achieving significant success [4]. It is evident that instantaneous HR can be estimated from a single cardiac cycle of the BVP signal. However, this estimation may exhibit significant deviations due to the noise and artifacts commonly present in rPPG-derived BVP signals. Accurately measuring average HR and heart rate variability (HRV) necessitates several cycles of stable BVP signals. Thus, the network must demonstrate high robustness to complex noise in order to extract BVP signals from shorter video segments. Additionally, the extracted BVP signals must be sufficiently clean and stable to ensure accurate heart rhythm measurements. Existing deep learning approaches for heart rhythm computation often require a substantial number of parameters and significant computational resources, which limits their applicability in real-time environments. Furthermore, these deep learning algorithms are susceptible to over-fitting due to the imbalance and variability in individual HR distributions under diverse conditions.

To address these challenges, we propose a lightweight network for measuring heart rhythm from facial videos, named MFF-Net, which is based on a multi-frequency mode fusion (MFF) mechanism. The main contributions are summarized as follows:

(1) A multi-frequency mode signal decomposition module based on empirical mode decomposition is introduced. This module enables the network to model characteristics separately across different frequency bands, effectively mitigating illumination variation and motion artifact disturbances while preserving the integrity of heart rhythm.
(2) A multi-frequency mode signal composition module is presented. This module utilizes a temporal multiscale convolution and a spectrum-based attention mechanism to effectively capture heart rhythm while optimizing computational resource usage.
(3) An over-sampling training scheme is implemented in MFF-Net. This approach aims to further reduce the risk of over-fitting in limited datasets.

## 2. Related Work

### 2.1. Unsupervised Models for Remote Photo-Plethysmography

The technology of extracting physiological information using cameras has developed rapidly in the last 10 years. Based on the physical principles of rPPG technology [5], several traditional noncontact HR measurement methods have been proposed. Among these, conventional blind source separation techniques, such as ICA [1] and time-domain filters (TDFs) [6], have shown effectiveness in extracting BVP signals in controlled environments. However, these techniques face significant limitations in real-world applications, particularly in processing nonlinear signals and handling noise. Consequently, the relatively low energy of the BVP signal, in comparison to various noise factors, makes the accurate extraction of the BVP a considerable challenge. To overcome these limitations, EMD was proposed to improve the algorithm’s efficiency in extracting BVP components from original rPPG signals [2], which captures the nonlinear characteristics and instantaneous frequency information of rPPG signals. However, it suffers from mode aliasing, which can compromise the accuracy of the results. In response to this challenge, methods such as ensemble empirical mode decomposition (EEMD) [7], variational mode decomposition (VMD) [8], and variational mode extraction (VME) [9] have been developed to address mode aliasing. Despite their advancements, these techniques typically require manual parameter tuning, which limits their adaptability to varying scenarios.

Some works focus on enhancing hardware performance by proposing multi-band cameras to obtain more robust BVP signals [10]. However, such solutions require advanced hardware and are challenging to implement on a large scale. Others have explored enhancements to filtering algorithms. For instance, Kim et al. [6] employed Bland-Altman and correlation analyses to process the original rPPG signals, while de Haan et al. [11] introduced a chromaticity-based color space projection (CHROM) to improve the quality of the rPPG signals. However, these methods are often affected by uncertainties in the mathematical models, leading to sub-optimal results when environmental disturbances deviate from the model assumptions.

### 2.2. Deep Learning Models for Remote Photo-Plethysmography

Extracting physiological information via deep learning has come a long way; it is divided into two main categories: HR calculating and BVP extraction. Radim et al. [12] used 3D convolution networks to process a video signal directly to calculate HR values. In previous work [13], a spatio-temporal representation was designed as the input of a CNN, which contains temporal and spatial features simultaneously. As a result, the stability and accuracy of HR estimation were significantly improved. These algorithms calculate HR directly, and although they work well, they are not able to extract BVP signals precisely, missing a lot of important information. Yu et al. [14] proposed a 3D spatio-temporal network for extracting BVP signals from video sequences, which firstly offered pearson correlation coefficients as the loss function for BVP signals extraction, significantly improving network performance. To recover rPPG signals from highly compressed facial videos, Yu et al. [15] proposed a video enhancement network in 2019. The next year, Yu et al. [16] presented a feature-extracting algorithm based on temporal difference convolution (TDC) using neural architecture search (NAS) for the first time. Niu et al. [17] designed a crossverified feature separation strategy to separate physiological features from nonphysiological features and then performed robust multitask physiological measurements using the extracted physiological features. Lu et al. [18] proposed a remote physiological measurement algorithm called DualGAN, jointly modeling the BVP signals predictor and noise distribution to suppress the noise mixed together with the physiological information. An advanced network architecture’s deep learning based method is able to perform better than traditional methods under normal illumination, yet one study [19] shows that it is not skilled in resisting fluctuating illuminations when facing a complex environment. Additionally, these deep learning methods demand substantial computational resources and require high-performance hardware for effective processing.

### 2.3. Attention Mechanism

Recently, there have been a number of works that incorporated attention processing to improve the performance of CNNs, which helps the network perform well regarding noisy inputs. Many existing, well-established attention mechanisms used in modeling in multiple dimensions have achieved tremendous success in classification tasks. Among them, the nonlocal network (NLNet) [20] pioneered the use of an algorithm to aggregate global context to each query location to capture long-term dependencies. Cao et al. [21] proposed the application of a simplified design similar to the squeezed excitation network (SENet) [22] in the above network structure, which can design a three-step generic framework for modeling global context more effectively. In addition, Woo et al. [23] created a lightweight generic module that performs adaptive feature refinement along the channel and spatial dimensions. The core construct in the well-known Transformer [24] network is the multi-headed self-attentive mechanism, which shows us that the self-attentive tool for extracting global features has great potential to improve algorithmic capabilities.

## 3. Method

**Figure 1:**
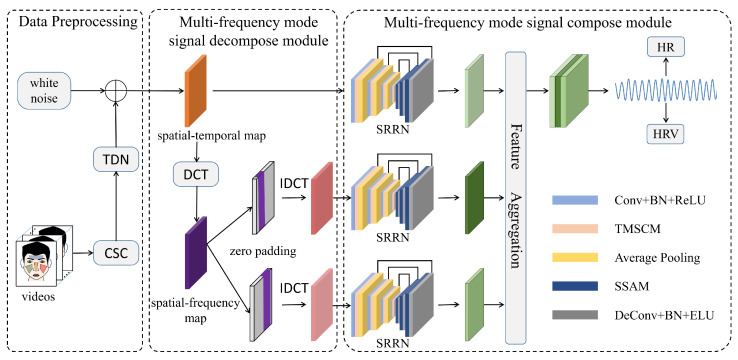
Overview of our MFF-Net.

In this section, we give detailed explanations of the proposed multi-frequency mode signal fusion mechanism for capturing BVP waveform information quickly from facial videos. Figure 1 gives an overview of the proposed method, including a data preprocessing module, a multi-frequency mode signal decompose module, a signal refinement and reconstruction network, and a multi-frequency mode signal compose module.

Firstly, the data preprocessing module cuts the facial image using landmark points localized by Mediapipe, which are then converted to the modified YUV color space (CSC) and normalized. This conversion, along with the application of time-domain normalization (TDN), serves to initially remove disturbances from the raw extracted signals. Additionally, white noise is introduced to simulate the environmental disturbances commonly present in real-world scenarios. This step is crucial for enhancing the model’s generalizability and adaptability. A detailed explanation of these processes is provided in Section 3.1. Next, the multi-frequency mode signal decomposition module extracts multi-frequency mode components from the spatio-temporal map in the frequency domain, obtaining multi-band signals. By decomposing the signal into the frequency domain, it becomes easier to identify and remove noise components, thereby enhancing the quality of the signal. Setting the frequency bands of interest serves as a method to balance physiological signals and noise signals, as discussed in Section 3.2.1. Finally, the multi-frequency mode signal composition module, which is based on the signal refinement and reconstruction network (SRRN), is designed to extract multiscale features and obtain the high-dimensional intrinsic mode signal. This module integrates features derived from multiple frequency components and outputs the target BVP. The detailed processes and methodologies involved in this composition are discussed in Section 3.2.2, and the details of our over-sampling training scheme are introduced in Section 3.3.

### 3.1. Data Preprocessing

The physiological information reflecting the blood flow is weak, which is usually contaminated by the disturbances induced by the illumination variation and head movements. Therefore, it is important to amplify the physiological component in facial videos. The data preprocessing part first processes the input video clip to obtain a spatio-temporal map. Specifically, Mediapipe [9] is used to perform landmark detection on the *i*th frame of the facial video, based on which the set of average pixel values of the four regions is obtained by cutting the facial regions. Then, color space conversion (CSC) is used to convert the spatio-temporal map to the modified YUV color space. The modified YUV gives more attention to brightness dimension features in the color space and reduces the noise caused by the difference in ambient illumination at different locations on the face. The modified color space can be formulated as

(1) $$\left[\begin{array}{c} Y(i)\\ U(i)\\ V(i) \end{array}\right]=\left[\begin{array}{ccc} 0.299 & 0.587 & 0.114\\ -0.169 & -0.331 & 0.5\\ 0.5 & -0.419 & -0.081 \end{array}\right]\left[\begin{array}{c} R(i)\\ G(i)\\ B(i) \end{array}\right]$$

where *i* indicates the average pixel of the *i*th facial region.

Subsequently, the time-domain normalization module (TDN) is applied to generate a normalized spatio-temporal map, which has been demonstrated to effectively reduce disturbances in numerous studies. Finally, white noise is added to the normalized spatio-temporal map during the training phase to simulate potential noise from environmental factors, such as ambient ilumination variations and motion artifacts, that are encountered in real-world scenarios.

### 3.2. Multi-Frequency Mode Signal Fusion Mechanism

This paper introduces a multi-frequency mode signal fusion mechanism to address the limitations of blind source separation algorithms, which require manual parameter tuning and are unable to effectively handle nonlinear rPPG signals affected by complex disturbances. Specifically, as shown in Figure 1, we use discrete cosine transform (DCT) and inverse discrete cosine transform (IDCT) to obtain the initial multi-band signals. Then, we exploit SRRN to extract multiscale features from each component of the multi-frequency mode signal, obtaining the high-dimensional intrinsic mode signal. Finally, by using the multi-frequency mode signal compose module, we obtain the target BVP signal by fusing the practical components of the high-dimensional intrinsic mode signal.

#### 3.2.1. Multi-Frequency Mode Signal Decompose Module

The reflected light intensity of each face region can reflect the intravascular substance concentration changes in this region, i.e., the characteristics of the different phases of the cardiac cycle, so we can consider the 1D signals of each color channel in each region of the face over a long period of time as individual BVP signals, and we then perform frequency domain operations on these initial BVP signals.

In order to extract multi-frequency mode components from the spatio-temporal map in the frequency domain, we set *K* frequency bands of interest and *I* facial regions; the whole process is shown in Algorithm 1.

**Algorithm 1:** Multi-frequency mode signal decompose module

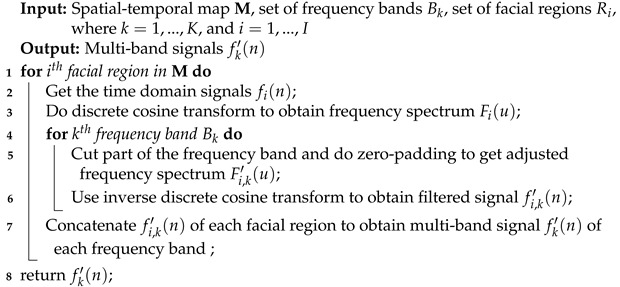



First, we extract the time-domain signal $f_{i}\left(n\right)$ for each facial region from the spatio-temporal map. The cosine discrete transform is then applied to each input time-domain signal, $f_{i}\left(n\right)$, to obtain $F_{i}\left(u\right)$. Based on the boundary defined by *K* frequency bands of interest, we cut part of the frequency spectrum and do zero-padding to obtain an adjusted frequency spectrum $F_{i,k}^{\prime }\left(u\right)$, which is then transformed into the filtered time domain signal $f_{i,k}^{\prime }\left(n\right)$ by using discrete cosine inverse transform. Finally, we concatenate the filtered time domain signals, $f_{i,k}^{\prime }\left(n\right)$, of each facial region and obtain the multi-band signals, $f_{k}^{\prime }\left(n\right)$, of each frequency band as an end result.

#### 3.2.2. Multi-Frequency Mode Signal Compose Module

In order to jointly model and extract clean BVP signals from the multi-frequency mode signal, a signal-refining and reconstruction network, which includes a signal-refinement network and a signal-reconstruction network, is proposed in this study.

To effectively extract signal features at multiple resolutions, we designed a signal-refinement network. The spatio-temporal map and the multi-band signal are fed into the network, and a corresponding high-dimensional frequency signal is the output. The pipeline of the signal-refinement network is designed as shown in Figure 1, named SRRN, which consists of a temporal multiscale convolution module (TMSC-module) convolution layer, an activation layer, a BN layer, and a pooling layer. It aims to gradually map the original signal to relatively pure potential stream shapes at lower resolutions, enhancing the network’s ability to adaptively filter out disturbance signals.

**Figure 2:**
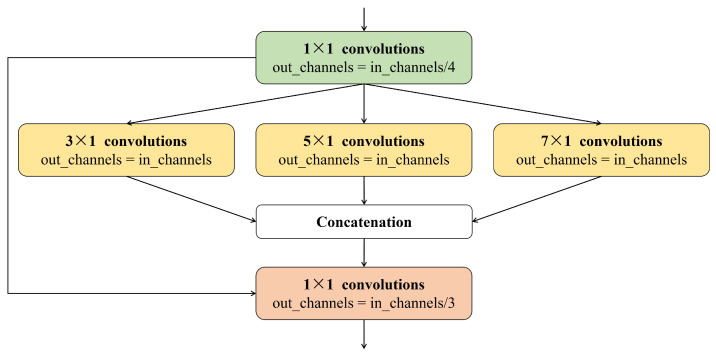
Schematic diagram of the temporal multiscale convolution module (TMSC-module).

In this network, the TMSC module is used as the main structure that can improve the network’s ability while keeping the computational budget constant. Moreover, in order to capture multiscale features in the time domain, we set the convolution kernel sizes of the TMSC-module to 3×1, 5×1, and 7×1. We concatenate the output of the three convolution blocks for fusing multiscale features and obtain a better representation to highlight the physiological information; the specific process is shown in Figure 2.

The signal reconstruction network, including the spectrum self-attention module (SSA-module), deconvolution layer, and activation and BN layer, uses the multiscale features in the feature extraction stage to perform the work of feature signal reconstruction. The main purpose of this design is to improve the SNR of the reconstructed signal and help the network locate the position of each peak more precisely.

We assume that the frequency domain features of different periods should be similar in a signal waveform. If there is a large difference in the waveform features in a certain period compared with other periods, we can confirm the presence of loud noise signals during this time. We can obtain the self-attention weights of each filter by using this mechanism and then put these weights into the channel attention module to globally group the individual filter features to further reduce the pulse wave noise.

The spectrum-based attention mechanism can be abstracted into three processes: (a) time-domain discrete cosine transform. The signal is segmented in the time domain and then subjected to discrete cosine transform to obtain the frequency features; (b) frequency domain self-attention, using 1×1 convolution to reduce the number of channels to 1D and extract the self-attention weight features for each period; (c) feature aggregation, using multi-convolution layers, combining global contextual features, and aggregating the original signal and self-attention weights to reconstruct the filtered signal. We fuse multiple high-dimensional intrinsic mode signals to extract the effective component and obtain the target BVP signal.

### 3.3. Over-Sampling Training Scheme

Due to the highly unbalanced distribution of training samples in terms of HR, using all samples directly in network training will inevitably over-fit a certain HR range. Therefore, for the network to effectively learn the BVP signals corresponding to all HR intervals, we first trained the network with all training samples using a large learning rate and then used a small learning rate to update the method with an over-sampling training scheme that divides the training samples into several groups according to the HR range. In each batch, the number of samples in each group is guaranteed to be in the same proportion, together with the data enhancement described in the data preprocessing module to maximize the limited training dataset and strengthen the network fitting ability.

## 4. Experiments and Results

### 4.1. Datasets and Experimental Settings

#### 4.1.1. Datasets

We evaluated our method on three widely used publicly available physiological measurement datasets, i.e., UBFC-rPPG [25], VIPL-HR [26], and MMSE-HR [27].

UBFC-rPPG comprises videos of 42 subjects, including 11 females and 31 males, engaging in a time-sensitive mathematical game under sunlight and indoor lighting conditions. It contains 42 RGB videos, all captured at 30 fps using a Logitech C920 HD Pro webcam, and corresponding actual BVP signals acquired using a CMS50E. In our experiments, UBFC-rPPG was used in the intra-dataset testing and ablation study. We trained the network on the first 30 subjects and tested the remaining 12 subjects.

VIPL-HR contains 2378 less-constrained RGB videos from 107 individuals, comprising 28 females and 79 males, aged from 22 to 41 years. The dataset encompasses a variety of scenarios, including nine different head movements and varying lighting conditions, and the videos were recorded using three different camera devices. Each individual contributed approximately 22 videos across these different scene-device combinations. The frame rate of all videos varies from 25 fps to 30 fps. Additionally, the corresponding HR and BVP signals are simultaneously recorded using the CONTEC CMS60C BVP sensor. We use this dataset for training in the crossdataset testing.

MMSE-HR is a large dataset of remote HR tests consisting of 102 videos taken from 40 individuals, including 23 females and 17 males, aged from 18 to 66 years. Each individual contributed approximately two to three videos. The dataset includes recordings of multiple facial expressions and head movements, all captured at a frame rate of 25 fps. The HR and physiological signals were collected using the Biopac MP150 data acquisition system. We used the MMSE-HR dataset for crossdataset testing.

#### 4.1.2. Evaluation Metrics

Various metrics were used for evaluation. For the task of average HR estimation, we used metrics including the standard deviation of the error (Std), the mean absolute error (MAE), the root mean squared error (RMSE), and Pearson’s correlation coefficients (r). For the task of HRV estimation, we followed existing methods [17,28] and used low-frequency (LF), high-frequency (HF), and LF/HF ratios in terms of Std, RMSE, and r.

### 4.2. Analysis of the Number of Parameters and FLOPs

We compared the network structure to five advanced deep learning methods, and the results are shown in the Table 1. The proposed MFF-Net method inputs 2D spatio-temporal maps of size 450 × 4 (with a video frame rate of 30 fps and a video segment length of 15 s). The multiscale spatio-temporal maps for CVD and DualGan were resized to 320 × 320 before being input into the network. The inputs for PhysNet, TSCAN, and EfficientPhys consist of raw RGB images of faces, which are located using Mediapipe, with a resolution adjusted to 128 × 128. Our method requires the least number of parameters, followed by CVD [17] and DualGAN [18]. Since the space size of the 2D spatio-temporal map we formed is 4, the network requires far fewer parameters than existing state-of-the-art algorithms. By only considering the testing phases, the related module of CVD includes the physiological encoder, Ep, nonphysiological encoder, En, the decoder, D, and the physiological estimator, the parameters of which are 42 k, and FLOPs = $1.1\times 10^{10}$. Similarly, the number of parameters in the BvpEstimator of DualGAN is approximately 66 k, and FLOPs = $9.9\times 10^{9}$. In contrast, the network that we provided contains only 11 k parameters in total, as shown in Table 1. The number of parameters is only one-fourth of CVD and one-sixth of DualGAN, which is sufficient to show that the network structure proposed in this paper is lightweight and has excellent advantages for cloud deployment. In terms of FLOPs, the FLOPs of the proposed network is $1.3\times 10^{8}$, which is smaller than the other five networks by an order of magnitude.

Moreover, if we consider the monitoring task of 30 s video, the proposed network in this paper can be completed by using only one calculation, but CVD can only calculate HR in 10 s intervals for each input, and it needs a total of 41 calculations to complete the whole task. The situation of DualGAN is similar. Therefore, both of the excellent existing methods use much higher computational complexity than our proposed network to complete the 30 s monitoring task.

### 4.3. Intra-Dataset Testing

#### 4.3.1. HR Estimation on UBFC-rPPG

We evaluated the average HR estimation on the UBFC-rPPG dataset. State-of-the-art methods, including four hand-crafted methods and seven deep learning based methods, were used for comparison. We can directly take the results of these state-of-the-art methods from previous work. The results of the proposed method and the state-of-the-art methods are given in Table 2. From the results, we can see that, in the case of a single HR calculation, the proposed method achieves promising results with an RMSE of 1.12 bpm, an MAE of 0.75 bpm, and an r of 0.99. It outperforms all the state-of-the-art traditional and deep learning methods, which need to obtain the average result of multiple overlapping video clips (except for Dual-GAN). This indicates that the BVP signal generated by our proposed algorithm is of higher quality and is a more accurate HR value that can be obtained from just one calculation.

#### 4.3.2. HRV Estimation on UBFC-rPPG

In following the protocol in [18], we used the first 30 subjects for training, and the remaining 12 subjects were used for testing. For the task of HRV estimation, we used several state-of-the-art methods, including POS [5], CHROM [11], Green [31], CVD [17], and DualGAN [18], for comparison, the results of which are taken from reference [18]. The HRV estimation results are presented in Table 3, where our proposed approach consistently outperforms all existing state-of-the-art methods across various metrics. These results demonstrate that our architecture can accurately recover the waveform shapes of BVP signals while utilizing fewer parameters, highlighting its efficiency. Importantly, our method not only calculates HR but also effectively estimates HRV, offering a comprehensive analysis of cardiovascular health. This dual capability facilitates a deeper understanding of physiological responses, thereby enhancing the overall utility of our approach for monitoring and assessing heart health.

### 4.4. Crossdataset Testing

In addition to the intra-dataset tests on the UBFC-rPPG dataset, following [17], we trained our method on VIPL-HR and tested it on MMSE-HR. The results of our proposed method and five other state-of-the-art methods, as reported in reference [18], are presented in Table 4, which illustrates that our proposed 30 s method significantly outperforms other state-of-the-art approaches. Specifically, when compared to the second-best method, CVD, our approach achieves an 8% reduction in Std, a 5% decrease in RMSE, and a 7% improvement in the correlation coefficient. These results highlight the strong generalization capabilities of our method in unconstrained scenarios, which are typically characterized by variable lighting and motion artifacts. Furthermore, the effectiveness of our multi-frequency mode signal decomposition and synthesis modules in addressing these challenges across diverse datasets emphasizes the robustness and universality of our approach, making it a valuable tool for real-world applications in physiological signal analysis.

### 4.5. Ablation Study

We also provide the results of ablation studies for the proposed method for HR estimation on the UBFC-rPPG dataset. All the results are shown in Table 5.

#### 4.5.1. Effectiveness of MFF Mechanism

From Table 5, we can see that when our network loses the MFF mechanism, the results fall off a cliff, with MAE dropping from 0.75 bpm to 2.35 bpm and RMSE and MAE dropping by 3.32 bpm and 1.6 bpm, respectively. The pyramid-like structure of the multiscale feature segmentation fusion mechanism allows the network to learn the high-dimensional features of the signal from multiple scale modes in all directions, taking into account both the global and local information of the signal. In addition, signals with complex noise are decomposed into multi-frequency modes, which contain less noise and are more traceable, validating the importance of our proposed MFF for improving the robustness of the network.

#### 4.5.2. Effectiveness of the Modified YUV

Table 5 demonstrates that the modified YUV color space enhances the effectiveness of the network to some extent when compared to the YUV color space. MAE drops from 2.05 bpm to 0.75 bpm and RMSE drops by 2.89 bpm. The Y channel in the modified YUV color space represents brightness, and the U and V channels represent chromaticity. Brightness refers to the degree of lightness or darkness of a color, which depends not only on the intensity of the light source but also on the reflection coefficient of the surface of the object. The variation in reflected light intensity in different stages of the heart cycle is reflected in the brightness of the face, so our method focuses more on brightness to improve the accuracy of the algorithm effectively.

#### 4.5.3. Effectiveness of Over-Sampling Training Strategy (OSS)

In order to validate the effectiveness of our over-sampling strategy, we trained our network with an over-sampling strategy as well as common sampling. From the results in Table 5, we can see that when we put all training set samples into network training in one epoch, the 30 s HR estimation results are worse than when using the over-sampling strategy. The MAE is reduced from 1.55 bpm to 0.75 bpm, and the RMSE is reduced from 3.13 to 1.12 bpm. This indicates that different heart rate intervals have similar temporal and spatial characteristics. Putting them equally into the network for training will help the network obtain better generalization performance and denoising ability regarding the whole heart rate interval.

#### 4.5.4. Effectiveness of the SSA-Module

We further evaluated the effectiveness of combining the SSA-module. The results in the Table 5 show that the robustness of the network is further enhanced by using the TMSC-module and SSA-module instead of the traditional residual convolution block, with MAE decreasing from 0.92 to 0.75 bpm and RMSE decreasing from 1.35 to 1.12 bpm. Our approach follows the attention mechanism-based approach but with several significant differences compared to existing methods: (1) In contrast to [21], which requires high-dimensional semantic features as the input, we convert the signal features into a spectrum in the time domain dimension, which is then fed into attention module. (2) Unlike [22], which lets the network automatically learn the weight of each dimension feature, we mainly locate the noise and calculate its weight by looking for the difference in signals in different periods. This validates our conception that the SSA-module can significantly improve the filtering ability of the physiological task against noise.

#### 4.5.5. Effectiveness of the TMSC-Module

We then evaluated the effectiveness of the TMSC-module. The results presented in Table 5 demonstrate that the multiscale convolution kernels within the TMSC-module significantly enhance the network’s time-domain perceptual field, enabling neurons to make decisions based on a richer set of information. By extracting multiscale features in the time domain, the TMSC-module contributes to improved estimation accuracy. Consequently, we observe a reduction in the MAE from 0.85 to 0.75 bpm and a decrease in the RMSE from 1.24 to 1.12 bpm. These improvements underscore the critical role of the TMSC-module in refining the model’s performance in physiological signal estimation.

## 5. Discussion

The accuracy of HRV extraction from short-duration facial videos serves as a key indicator of the network’s signal processing capabilities. To rigorously assess this aspect, we specifically designed a 15 s HRV prediction experiment. This experiment aimed to validate the superiority of our proposed method by demonstrating its effectiveness in accurately capturing and analyzing HRV from brief video segments. The results of this experiment not only highlight the robustness of our approach but also underscore its potential for real-time applications in physiological monitoring.

In the HRV estimation task, our proposed 15 s approach demonstrates exceptional performance across three key metrics, achieving results that only slightly trail those of DualGAN, as shown in Table 3. This outcome directly confirms the capability of MFF-Net to accurately predict the waveform of physiological signals. Notably, our heart rate results are derived from the BVP signal waveform rather than relying on a separate HR estimation module. This reliance necessitates that our network extracts signals of the highest quality, characterized by clean waveforms with minimal noise, to ensure precise calculations. Consequently, these findings further validate the effectiveness of our algorithm in reconstructing the BVP signal, highlighting its robustness in practical applications.

In addition, we also used crossvalidation to evaluate our proposed short-time method for estimating HR on MMSE-HR. From Table 4, we can see that the proposed approach achieves a promising result with an RMSE of 6.61 bpm when only using 15 s facial video. Compared with other existing 30 s methods, only the result of CVD is better than ours. The results on multiple datasets for different physiological measurement tasks show that our MFF-Net is effective at representing physiological information from face videos within 15–30 s.

Currently, the public datasets utilized in our study primarily consist of participants with healthy physiological profiles, which may limit the generalizability of our model. However, prior research has demonstrated that rPPG technology can effectively capture and reflect arrhythmias, particularly in the context of atrial fibrillation detection [36,37,38]. This capability highlights the need to integrate a broader spectrum of physiological conditions into our dataset. Consequently, we plan to collect additional data from individuals exhibiting arrhythmias and other health conditions in future studies. By enriching the diversity of our data distribution, we aim to enhance the robustness and adaptability of our model for complex physiological signals, enabling its effective application across a broader range of real-world scenarios. To further this goal, we are also collecting additional electrocardiography (ECG) signals, which will enhance the model’s capability to recover intricate physiological patterns. These strategic approaches will not only deepen our understanding of arrhythmia-related variations but also foster the development of more versatile applications in both clinical and nonclinical settings.

## 6. Conclusions

This study proposes a novel method for the fast and stable computation of heart rhythm at the cost of low computational resources. We first designed a multi-frequency mode signal fusion mechanism to suppress illumination variations and motion artifact disturbances, enabling the rapid acquisition of stable BVP signals. Next, we proposed a spectrum-based attention module (SSA-module), which automatically locates specific locations of high noise in spatio-temporal features, and a powerful temporal multiscale convolution module (TMSC-module) with temporal difference convolution based on 2D spatio-temporal features, intending to capture BVP signal information using limited computational and time budgets. Finally, we developed an over-sampling training strategy to solve the over-fitting phenomenon. The MFF and over-fitting sampling scheme proposed in this paper can also be applied to other signal processing tasks.

## Figures and Tables

**Table 1:** The number of parameters and FLOPs.

Method	Params (MB)	FLOPs
PhysNet [14]	0.77	$4.5\times 10^{11}$
TSCAN [29]	0.75	$4.1\times 10^{9}$
EfficientPhys [30]	2.16	$1.8\times 10^{10}$
CVD [17]	0.42	$1.1\times 10^{10}$
DualGAN [18]	0.66	$9.9\times 10^{9}$
MFF-Net (Ours)	0.11	$1.3\times 10^{8}$

Notes: Bold indicates best performance

**Table 2:** HR estimation results of our method and several state-of-the-art methods on the UBFC-rPPG dataset.

Method	MAE↓	RMSE↓	r↑
CHROM [11]	8.20	9.92	0.27
POS [5]	8.35	10.00	0.24
Green [31]	6.01	7.87	0.29
Li2014 [32]	19.87	10.20	0.56
PhysNet [14]	1.15	3.70	0.94
TSCAN [29]	1.34	5.41	0.90
EfficientPhys [30]	1.64	5.53	0.89
PulseGAN [33]	1.19	2.10	0.98
SynRhythm [34]	5.59	6.82	0.72
And-rPPG [35]	3.15	4.75	0.92
Dual-GAN [18]	0.44	0.67	0.99
MFF-Net (ours)	*0.75*	*1.12*	*0.99*

Notes: Bold indicates best performance; italic indicates second-best performance; ↑ symbol indicates that larger values are better; ↓ symbol indicates that smaller values are preferred.

**Table 3:** The results of HRV analysis for the proposed approach and several state-of-the-art methods on the UBFC-rPPG dataset.

Method		LF-(u.n)			HF-(u.n)			LF/HF	
	Std↓	RMSE↓	r↑	Std↓	RMSE↓	r↑	Std↓	RMSE↓	r↑
POS [5]	0.171	0.169	0.479	0.171	0.169	0.479	0.405	0.399	0.518
CHROM [11]	0.243	0.240	0.159	0.243	0.240	0.159	0.655	0.645	0.226
Green [31]	0.186	0.186	0.280	0.186	0.186	0.280	0.405	0.399	0.518
CVD [17]	0.053	0.065	0.740	0.053	0.065	0.740	0.169	0.168	0.812
Dual-GAN [18]	*0.034*	*0.035*	*0.891*	*0.034*	*0.035*	*0.891*	*0.131*	*0.136*	*0.881*
MFF-Net (30 s)	0.030	0.030	0.921	0.030	0.031	0.921	0.101	0.101	0.895
MFF-Net (15 s)	0.041	0.042	0.850	0.041	0.042	0.85	0.143	0.143	0.853

Notes: Bold indicates best performance; italic indicates second-best performance; ↑ symbol indicates that larger values are better; ↓ symbol indicates that smaller values are preferred.

**Table 4:** The cross dataset HR estimation results for the proposed approach and several state-of-the-art methods on the MMSE-HR dataset.

Method	Std↓	RMSE↓	r↑
Li2014 [32]	20.02	19.95	0.38
CHROM [11]	14.08	13.97	0.55
SAMC [27]	12.24	11.37	0.71
RhythmNet [13]	6.98	7.33	0.78
CVD [17]	*6.06*	*6.04*	*0.84*
MFF-Net (30 s)	**5.60**	**5.75**	**0.90**
MFF-Net (15 s)	6.57	6.61	0.86

Notes: Bold indicates best performance; italic indicates second-best performance; ↑ symbol indicates that larger values are better; ↓ symbol indicates that smaller values are preferred.

**Table 5:** Ablation study of our MFF-Net in terms of OSS, TMSC, SSA, and MFF for HR estimation on UBFC-rPPG.

Method	MAE↓	RMSE↓	r↑
Without MFF	2.35	4.56	0.91
Without modified YUV	2.05	4.01	0.96
Without OSS	1.55	3.13	0.92
Without SSA	0.92	1.35	0.98
Without TMSC	0.85	1.24	0.98
MFF-Net	**0.75**	**1.12**	**0.99**

Notes: Bold indicates best performance; ↑ symbol indicates that larger values are better; ↓ symbol indicates that smaller values are preferred.

## Data Availability

All data are available from the corresponding author upon reason able request. And the original contributions presented in the study are included in the article, further inquiries can be directed to the corresponding authors.

## References

[1] Macwan R., Benezeth Y., Mansouri A. (2019). Heart rate estimation using remote photoplethysmography with multi-objective optimization. Biomed. Signal Process. Control.

[2] Boda S., Mahadevappa M., Dutta P.K. (2021). A hybrid method for removal of power line interference and baseline wander in ECG signals using EMD and EWT. Biomed. Signal Process. Control.

[3] Zheng X., Zhang C., Chen H., Zhang Y., Yang X. (2022). Remote measurement of heart rate from facial video in different scenarios. Measurement.

[4] McDuff D. (2023). Camera measurement of physiological vital signs. ACM Comput. Surv..

[5] Wang W., den Brinker A.C., Stuijk S., de Haan G. (2017). Algorithmic principles of remote PPG. IEEE Trans. Biomed. Eng..

[6] Kim B., Yoo S. (2006). Motion artifact reduction in photoplethysmography using independent component analysis. IEEE Trans. Biomed. Eng..

[7] Yin R.N., Jia R.S., Cui Z., Yu J.T., Du Y.B., Gao L., Sun H.M. (2021). Heart rate estimation based on face video under unstable illumination. Appl. Intell..

[8] Das M., Choudhary T., Bhuyan M.K., Sharma L.N. (2022). Non-contact heart rate measurement from facial video data using a 2D-VMD scheme. IEEE Sensors J..

[9] Liu B., Zheng X., Ivan Wu Y. (2024). Remote heart rate estimation in intense interference scenarios: A white-box framework. IEEE Trans. Instrum. Meas..

[10] Wang W., den Brinker A.C., Stuijk S., de Haan G. (2017). Robust heart rate from fitness videos. Physiol. Meas..

[11] de Haan G., Jeanne V. (2013). Robust pulse rate from chrominance-based rPPG. IEEE Trans. Biomed. Eng..

[12] Irani R., Nasrollahi K., Moeslund T.B. Improved pulse detection from head motions using DCT. Proceedings of the 2014 International Conference on Computer Vision Theory and Applications (VISAPP).

[13] Niu X., Shan S., Han H., Chen X. (2020). RhythmNet: End-to-end heart rate estimation from face via spatial-temporal representation. IEEE Trans. Image Process..

[14] Yu Z., Li X., Zhao G. Remote photoplethysmograph signal measurement from facial videos using spatio-temporal networks. Proceedings of the Computer Vision and Pattern Recognition.

[15] Yu Z., Peng W., Li X., Hong X., Zhao G. Remote heart rate measurement from highly compressed facial videos: An end-to-end deep learning solution with video enhancement. Proceedings of the 2019 IEEE/CVF International Conference on Computer Vision (ICCV).

[16] Yu Z., Li X., Niu X., Shi J., Zhao G. (2020). AutoHR: A strong end-to-end baseline for remote heart rate measurement with neural searching. IEEE Signal Process. Lett..

[17] Niu X., Yu Z., Han H., Li X., Shan S., Zhao G. (2020). Video-based remote physiological measurement via cross-verified feature disentangling. Proceedings of the Computer Vision—ECCV 2020: 16th European Conference.

[18] Lu H., Han H., Zhou S.K. Dual-GAN: Joint BVP and noise modeling for remote physiological measurement. Proceedings of the 2021 IEEE/CVF Conference on Computer Vision and Pattern Recognition (CVPR).

[19] Yang Z., Wang H., Lu F. (2022). Assessment of Deep Learning-Based Heart Rate Estimation Using Remote Photoplethysmography Under Different Illuminations. IEEE Trans. Hum.-Mach. Syst..

[20] Wang X., Girshick R., Gupta A., He K. Non-local neural networks. Proceedings of the IEEE Conference on Computer Vision and Pattern Recognition (CVPR).

[21] Cao Y., Xu J., Lin S., Wei F., Hu H. GCNet: Non-local networks meet squeeze-excitation networks and beyond. Proceedings of the IEEE/CVF International Conference on Computer Vision (ICCV) Workshops.

[22] Hu J., Shen L., Sun G. Squeeze-and-excitation networks. Proceedings of the IEEE Conference on Computer Vision and Pattern Recognition.

[23] Woo S., Park J., Lee J.Y., Kweon I.S. CBAM: Convolutional block attention module. Proceedings of the European Conference on Computer Vision (ECCV).

[24] Vaswani A., Shazeer N., Parmar N., Uszkoreit J., Jones L., Gomez A.N., Kaiser Ł., Polosukhin I. Attention is all you need. Proceedings of the Advances in Neural Information Processing Systems.

[25] Bobbia S., Macwan R., Benezeth Y., Mansouri A., Dubois J. (2019). Unsupervised skin tissue segmentation for remote photoplethysmography. Pattern Recognition Letters..

[26] Niu X., Han H., Shan S., Chen X., Jawahar C., Li H., Mori G., Schindler K. (2019). VIPL-HR: A multi-modal database for pulse estimation from less-constrained face video. Proceedings of the Computer Vision—ACCV 2018.

[27] Tulyakov S., Alameda-Pineda X., Ricci E., Yin L., Cohn J.F., Sebe N. Self-Adaptive Matrix Completion for Heart Rate Estimation from Face Videos under Realistic Conditions. Proceedings of the 2016 IEEE Conference on Computer Vision and Pattern Recognition (CVPR).

[28] Tang C., Lu J., Liu J. Non-contact heart rate monitoring by combining convolutional neural network skin detection and remote photoplethysmography via a low-cost camera. Proceedings of the 2018 IEEE/CVF Conference on Computer Vision and Pattern Recognition Workshops (CVPRW).

[29] Liu X., Fromm J., Patel S., McDuff D. Multi-task temporal shift attention networks for on-device contactless vitals measurement. Proceedings of the 34th Conference on Neural Information Processing Systems.

[30] Liu X., Hill B., Jiang Z., Patel S., McDuff D. EfficientPhys: Enabling simple, fast and accurate camera-based cardiac measurement. Proceedings of the 2023 IEEE/CVF Winter Conference on Applications of Computer Vision.

[31] Verkruysse W., Svaasand L., Nelson J. (2008). Remote plethysmographic imaging using ambient light. Opt. Express.

[32] Li X., Chen J., Zhao G., Pietikäinen M. Remote Heart Rate Measurement from Face Videos under Realistic Situations. Proceedings of the 2014 IEEE Conference on Computer Vision and Pattern Recognition.

[33] Song R., Chen H., Cheng J., Li C., Liu Y., Chen X. (2021). PulseGAN: Learning to Generate Realistic Pulse Waveforms in Remote Photoplethysmography. IEEE J. Biomed. Health Inform..

[34] Niu X., Han H., Shan S., Chen X. SynRhythm: Learning a deep heart rate estimator from general to specific. Proceedings of the 2018 24th International Conference on Pattern Recognition (ICPR).

[35] Lokendra B., Puneet G. (2022). AND-rPPG: A novel denoising-rPPG network for improving remote heart rate estimation. Comput. Biol. Med..

[36] Wu B.F., Wu B.J., Cheng S.E., Sun Y., Chung M.L. (2023). Motion-robust atrial fibrillation detection based on remote-photoplethysmography. IEEE J. Biomed. Health Inform..

[37] Tseng C.W., Wu B.F., Yu S. (2024). A real-time contact-free atrial fibrillation detection system for mobile devices. IEEE J. Biomed. Health Inform..

[38] Li X., Alikhani I., Shi J., Seppanen T., Junttila J., Majamaa-Voltti K., Tulppo M., Zhao G. The OBF database: A large face video database for remote physiological signal measurement and atrial fibrillation detection. Proceedings of the 2018 13th IEEE International Conference on Automatic Face and Gesture Recognition (FG 2018).

